# Institutional Notes and News

**Published:** 1911-03-04

**Authors:** 


### Institutional Notes and News.

**Bradford Children's Hospital.**

Mr. J. R. Jeffery, at the annual meeting of the Bradford Children's Hospital, lamented the fact that there was little chance for this institution to participate in the funds now being raised for a local memorial to the late King. He was afraid "the claim which had been put forward by the Bradford Royal Infirmary for practically sole recognition as the King's memorial for the city had shut the door on any united action." Last week, however, we were glad to note the starting of a County or West Riding Fund "the object of which is to provide money for the local hospitals as memorials in more than one place." We hope that this will notice the claims of the Children's Hospital. Here, at any rate, is a summary of its past year's work: 602 in-patients, or a daily average of 56.4, received treatment, and 2,627 persons were treated as out-patients; in other words, an increase under both these headings. The total income for the year was £2,986, a slight improvement, the main factor in which was the big advance of £118 from the Bradford Hospital Sunday Fund. There is a debt of £789 on the year, and £829 is still due on the out-patients' building account. Mr. A. T. Parkinson, the hon. secretary, has here all the material for a good case.

**Forster Green Hospital, Belfast.**

Professor H. O. Meredith made some interesting remarks on the national problem of consumption at the recent annual meeting of the Forster Green Hospital, Belfast. He said that by taking thought we could add inches to our stature, and, as it was established that the economic prosperity of nations was based upon a vigorous condition of family life, the next generation must be cared for with adequate nourishment, pleasure, and housing. The peculiar cruelty, he added, about tuberculosis was that it often cut down the wage-earner who kept the home together. The number of patients treated had risen during the past year to 337, and each remained on an average for twelve weeks in the hospital. On discharge "in 66 patients the disease was arrested, 148 were improved, 24 were not improved, and two died in hospital." We hope that the after-histories of patients are carefully preserved, as it is on these alone that general statistics of any value can be based. A home for advanced cases, who tend to fill the existing sanatoria to the disadvantage of incipient consumptives, or else have nowhere to go, is badly wanted in the city.

**Charing Cross Appeal.**

Lady Juliet Duff is conducting her appeal on behalf of the Charing Cross Hospital with capital persistence and originality. Most people in the neighbourhood of Charing Cross will have received her letter, and many of them have received already a first-hand account of why the appeal is being made, and of the financial difficulties, by the representatives, or rather the ambassadors, who are bringing the hospital's case straight into the homes of the neighbouring public. This direct method should have its reward, and Mr. Walter Alvey should share with the hospital's treasurer a feeling of relief that the mortgage debt by which Charing Cross Hospital is hampered must be in process at least of liquidation. If the Strand could be made to realise for five minutes the work done at the hospital which watches over its traffic the debt on the mortgage would be paid off at once. It has guarded the Strand for over seventy years. it has grown up with the increasing crowds which fill it, and one of the richest streets in the city surely cannot be appealed to in vain.

**Worcester Ophthalmic Hospital.**

The Annual Meeting of the Worcester Ophthalmic Hospital was held under the presidency of Lord Lifford. The report showed a surprising increase in work, largely the result of the inspection of the school children in the city and county; and Mr. Francis Wood, the Chairman of Committee, expressed the hope that some grant might be made by the County Council, in consideration of the large number of children from country districts who were treated at the Institution. The in-patients numbered 72 as compared with 59. Perhaps on this account the balance sheet did not present so encouraging a record. The income for the year was £496 as against £563, while the expenditure was £548 16s. 8d. as against £537 13s. 11d. The deficit of £35 with which the year had begun was then increased to £88. These notes continually have to record the more or less serious deadlocks which are occurring all over the country on the vexed question of medical inspection, and its effects on the hospital. At Hereford and now at Liverpool resolutions have been passed by the medical men, asking respectively for some form of medical State service, or for a grant in aid.

**Royal Children's Hospital, Glasgow.**

The rebuilding of the Royal Hospital for Sick Children, Glasgow, is likely now to proceed without further let. It will be remembered that the original plans provided for a hospital on the pavilion system, each block of which was to have been of two storeys. On account of the cost this has been abandoned, unfortunately, as all the hospital's supporters must feel; and a complete remodelling of the plans followed, the aim of which was to reduce the number of pavilions and to add to the height of each. The accommodation has been cut down from 300 to 200 cots, and of the ten acres of the York Hill site, less ground will be occupied under the new scheme. The cost was expected to be £120,000, and will prove more than that, but it is encouraging to think that £102,000 has been subscribed towards it. In the meantime work in what will soon be the old hospital is proceeding: 997 patients were treated in the wards last year, and the daily average number was 67. The county branch at Drumchapel has been continuously full, and more than the 26 cots could have been occupied. The income for the past year has increased by nearly £4,000, while the expenditure has shown practically no change.

**Leicester Infirmary.**

The annual statement of accounts to be presented at the 138th anniversary meeting (at which the Duke of Rutland will preside) shows a total income of £21,531 3s., £1,355 8s. 11d. of which is from legacies and special donations. The receipts from the Saturday Hospital Fund once more show a substantial advance, £13,791 4s. 3d. being recorded, seven-tenths of which, less expenses, is devoted to the infirmary, amounting to £9,366 3s. 7d. The annual ordinary expenditure is £20,914 3s. 1d., and the total expenditure £21,071 6s. After making provision for certain sums required to be invested (£248 19s.), and carrying forward £150 to reserve fund for contingent improvements, a balance of £60 18s. remains in hand. The average cost per in-patient amounts to £5 15s. 4d. and the cost per bed occupied to £83 9s. 9d. The average cost per out-patient is 2s. 2d.

**Aberystwyth Infirmary and Cardiganshire General Hospital.**

The President, Captain Cosens, took the chair at the recent annual meeting of Governors and Subscribers. During the year 161 in-patients were treated and 1,004 out-patients. Owing to a falling off in special collections the receipts were considerably less than in the previous year, and there was a deficiency on the year's working of £167 2s. It was announced by the Chairman that Sir John Williams had consented to become a consulting physician to the Institution. During the year a much-needed improvement was carried out, namely, the installation of heating apparatus right through the building. The operating-room has been renovated, and is now laid out and furnished on modern scientific principles. The *x*-ray apparatus which was installed three years ago has proved a great boon.

**Worcester General Infirmary.**

At the monthly meeting of the Executive Committee a letter was received from the Board of Guardians asking to be allowed to appoint one of their members to sit on the Infirmary Committee. It was decided to call the attention of the Board to the rule which directs that the Committee shall be elected by the Governors. A letter was read from the secretary of the Hospital Saturday Committee asking that the words on the Infirmary tickets referring to "charity" be altered, suggesting the formula in use at the Birmingham General Infirmary, to the effect that the applicant "is a person whom I believe to be a proper subject for relief at the Institution" in their stead. After much discussion, a resolution to the effect that the wording of the tickets objected to should be altered to the following: "a person whom I believe to be deserving of the benefits of the Infirmary" was carried, some members abstaining from voting.

**Glasgow Royal Infirmary.**

A new development in hospital policy which may be the beginning of a comprehensive change in the administration of hospitals in general was recorded at the annual meeting of the Glasgow Royal Infirmary. This is the appointment of a joint consultative committee for the three infirmaries of the city. Speaking on this subject at the above meeting, Lord Provost M'Innes Shaw is reported to have said: "Some time ago I took occasion to urge the desirability of some concentrated scheme of administration being introduced in connection with the work of your three great infirmaries, and I am happy to know that my words on that occasion have borne fruit by the establishment during the year of a joint consultative committee consisting of representatives from each of the Royal, Western, and Victoria Infirmaries. This arrangement cannot but operate to the highest advantage of all the institutions concerned, as there are numerous matters of mutual interest regarding which it will be to the public advantage that joint and uniform action shall be taken." The Royal Infirmary and the University have also collaborated, so that new arrangements for medical teaching have been entered into between the two. In this way the unique field for study which the Infirmary offers will be made use of to the full. The number of admissions has increased, with the result that there is an overdraft owing to the accompanying rise in the hospital's expenditure.

